# Belugas (*Delphinapterus leucas*) create facial displays during social interactions by changing the shape of their melons

**DOI:** 10.1007/s10071-024-01843-z

**Published:** 2024-03-02

**Authors:** Justin T. Richard, Isabelle Pellegrini, Rachael Levine

**Affiliations:** Department of Fisheries, Animal and Veterinary Science, University of Rhode Island, Kingston, RI 02881 USA

**Keywords:** Cetacean, Beluga, Gestural communication, Facial display

## Abstract

Beluga whales are considered unique among odontocetes in their ability to visibly alter the appearance of their head by changing the shape of the melon, but only anecdotal observations are available to evaluate the use or potential function of these melon shapes. This study of belugas in professionally managed care aimed to establish an ethogram for the repertoire of categorizable melon shapes and then evaluate their potential function as intentional communication signals by determining if they were produced and elaborated during social interactions of varying behavioral contexts while in the line of sight of a recipient. Five different melon shapes were reliably identified in video observations of the primary study population (*n* = 4) and externally validated in a second aquarium population (*n* = 51). Among the 2570 melon shapes observed from the primary study subjects, melon shapes occurred 34 × more frequently during social interactions (1.72 per minute) than outside of social interactions (0.05 per minute). Melon shapes occurring during social interactions were performed within the line of sight of a recipient 93.6% of the time. The frequency of occurrence of the different melon shapes varied across behavioral contexts. Elaboration of melon shapes through extended duration and the occurrence of concurrent open mouth displays varied by shape type and across behavioral contexts. Melon shapes seem to function as visual displays, with some characteristics of intentional communication. This ability could yield adaptive benefits to belugas, given their complex social structure and hypothesized mating system that emphasizes pre-copulatory female mate choice.

**Supplementary Information:**

The online version contains supplementary material available at 10.1007/s10071-024-01843-z.

## Introduction

**Fig. 1:**
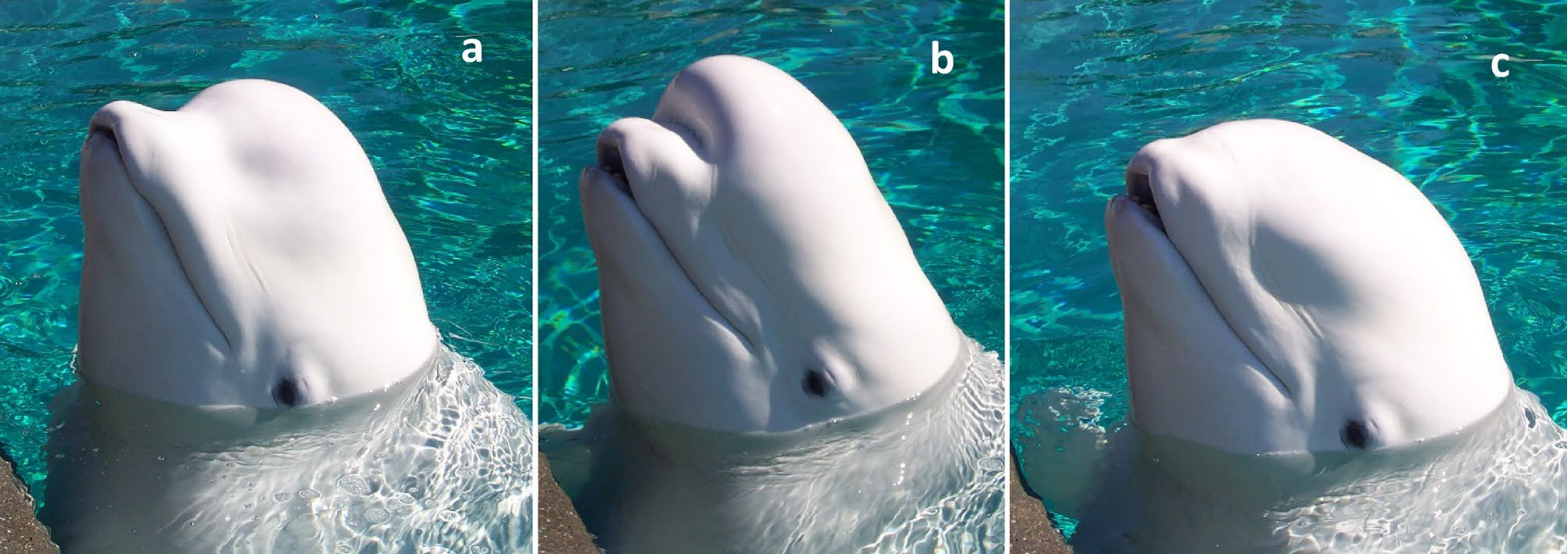
A trained beluga demonstrating the ability to voluntarily change the shape of the melon from the relaxed position (**a**), to extended rostrally (**b**) and retracted posteriorly (**c**)

Facial expressions and displays are an important mode of communication in many mammals. They provide recipients with information about the actor’s affective state (Kret et al. 2020), immediate intentions (Lazow and Bergman 2020), or help convey the meaning of signals used in multimodal displays (Aychet et al. 2021). Facial expressions occur across a wide variety of mammalian taxa, being best studied in primates (Waller and Micheletta 2013), but they have also been studied in dogs (Kaminski et al. 2017), pigs (Mota-Rojas et al. 2020), and horses (Wathan et al. 2016). However, contrary to most mammals, odontocete cetaceans are largely incapable of facial expressions due to the relative immobility of the areas of the eyes and mouth (Mead 1975). Although facial muscles are present in odontocetes, the bottlenose dolphin’s (*Tursiops truncatus*) face has been described as an “expressionless mask” (Cozzi et al. 2016). The exception among odontocetes is the beluga. When describing beluga natural history for scientific purposes or the lay public, authors frequently cite their ability to create facial expressions, primarily by changing the shape of their melon, the anatomical structure composed of lipid situated in the forehead region of odontocetes (Fig. 1; e.g. Brodie 1989; O’Corry-Crowe 2018). While commonly referred to as facial expressions, it is unknown if these melon conformation changes (hereafter “shapes”) are categorizable into discrete behaviors, and if so, if they have a communicative function. If they do function as communication signals, melon shapes may be reflexively produced in response to changes in affect like some primate facial expressions, or may be more accurately described as intentionally produced gestures or displays. Given their apparently unique nature, evaluating the function of these melon shapes could yield new insights into beluga behavioral ecology.

The melon is an important anatomical feature for echolocating odontocetes, primarily functioning to transmit echolocation clicks into the water while directing these clicks forward (Cranford et al. 1996; Harper et al. 2008; McKenna et al. 2012). Despite this conserved function among odontocetes, variation in the morphology of the melon, associated muscles and connective tissue creates a variety of head shapes, from the bulbous shape of the pilot whale (*Globicephala sp.*) to the narrow, blunt shape of the harbor porpoise (*Phoceona phocoena*) (Harper et al. 2008; Mead 1975). Melon shape may influence the echolocation beam, with longer melons allowing greater focusing ability, and wider melons enabling the use of clicks with a wider frequency range (McKenna et al. 2012). The beluga’s melon is particularly bulbous, and the large head allows the production of a narrower, more directional echolocation beam than in bottlenose dolphins (Au et al. 1987). The ability to change the shape of the melon may therefore confer further benefits to an echolocating odontocete. While all odontocetes have facial muscles associated with the melon that are hypothesized to change the density or shape of the melon (Harper et al. 2008; McKenna et al. 2012), only belugas have been documented to visibly alter the shape of their head. Hereafter, we will use “melon” to refer to the entire mobile region of the beluga’s head. Unlike the case for other species (*Tursiops truncatus*, Harper et al. 2008; *Phocoena phocoena*, Huggenberger et al. 2009; *Kogia sima* and *Kogia breviceps*, Thornton et al. 2015; see also Mead 1975 for a brief overview of facial musculature in 10 other odontocete species), a detailed description of the beluga facial musculature, and thus an anatomical explanation for their unique facial mobility, is unavailable.

Despite the theoretical acoustic benefits, the beluga’s melon shape changes have largely been observed in social contexts. In an ethogram developed from belugas in managed care, DiPaola (2007) defines “melon extensions” that occur in aggressive contexts, in which the actor “markedly changes the shape of the melon, forming a ball and pushing it forward.” Hill et al. (2015b) observed “melon thrusts” during agonistic interactions between belugas in managed care but did not define this behavior. Krasnova et al. (2014) state that the melon becomes “enlarged” in “excited” male belugas observed in the White Sea, implying that females were not observed performing this behavior. Although often documented, a rigorous ethogram has not been created for beluga melon shapes that could correlate to the facial musculature of odontocetes, and only anecdotal observations have been performed without attempts to further quantify the occurrence of these behaviors. One exception is the melon shake, in which the actor vigorously shakes the head in dorsal/ventral plane, causing apparently passive yet marked extension and compression of the melon. Richard et al. (2021) quantified the occurrence of this behavior and found that it was primarily performed by males toward a female recipient in conjunction with courtship behavior. These seemingly context-specific observations suggest a social function for melon shapes in belugas, analogous to facial expressions or displays, instead of a purely acoustic function.

If melon shapes have a communicative function, then exploring the context in which these displays are produced may provide evidence of the intended signals these displays may communicate. For example, Aychet et al. (2021) used the occurrence of specific behaviors performed by red-capped mangabeys (*Cercocebus torquatus*) to characterize behavioral contexts such as playful, affiliative, or agonistic interactions that included facial displays to demonstrate context dependent performance of several displays. Similarly, detailed observations of belugas in aquaria, where the animals can be observed underwater and from close proximity, have identified behavioral indicators of various contexts, including courtship (male toward female genital presents during ovarian cycling, Richard et al. 2021), socio-sexual behavior (male-male genital presents, Hill et al. 2015b; Richard et al. 2021), agonistic behavior (biting; Hill et al. 2015b), and affiliative behavior (group swimming, Hill et al. 2015b; Richard et al. 2021). While establishing the goals of a communication signal in nonhuman animals is challenging, context specific use of melon shapes would support their communicative function.

If melon shapes are communication signals, then determining if melon shapes are reflexive actions or intentionally produced would further define the potential functions of these behaviors. Intentional communication is a foundation of human language, and therefore studying this capacity in nonhuman animals is important in understanding the evolution of language (Townsend et al. 2017). For communication to be considered intentional, the signal must be goal directed behavior, voluntarily produced by a signaler toward a receiver in order to achieve the goal, and result in a behavioral change in the receiver that is consistent with the goals of the signaler (Townsend et al. 2017). While nonhuman primate gestures are largely considered intentional communication, more recently facial expressions, which are typically considered reflexive responses to emotional state, have been shown to have characteristics of intentional communication; these facial movements are thus referred to as facial gestures or displays (Aychet et al. 2021; Molesti et al. 2020; Waller et al. 2015). These studies evaluated whether facial displays occurred in a social context and within the view of a recipient as behavioral measures of the voluntary production of the signal, one of the characteristics of intentional communication. Further evidence of intentionality comes from the elaboration of a display, for example by varying the intensity of the display (Waller et al. 2015), or by repeating or pairing the display with other displays (Roberts et al. 2014). Belugas in human care can be trained to change the shape of the melon on cue (Fig. 1), demonstrating that they have voluntary control of the muscles responsible for creating melon shapes. The use of melon shapes primarily during social interactions, in the view of a recipient, with varied elaboration, would further support a role in visual communication.

By observing a group of belugas under professionally managed care in an aquarium with extensive underwater viewing, this study aims to establish an ethogram of melon shapes that can be used to quantify discrete behaviors. The repertoire of melon shapes is predicted to be similar for each individual, reflecting underlying facial musculature found in odontocetes. Then, using this ethogram, the frequency and behavioral context of these melon shapes will be determined for these belugas. Based on existing information suggesting a social function of these behaviors as well as their ability to voluntarily control the associated musculature, melon shapes are predicted to occur primarily during social interactions within the field of view of a recipient, and the types of shapes are predicted to vary by social context. Melon shapes are predicted to be elaborated through varying intensity, as measured by the duration of the shape, and with the varying simultaneous occurrence of other display behaviors. This elaboration is predicted to vary by shape type and behavioral context. Alternatively, if melon shapes are used solely for echolocation purposes, we predict the shapes would occur at similar frequencies during and outside of social contexts, and those occurring during social interactions would be performed with similar frequency by different actors and would not vary in frequency or elaboration across behavioral contexts.

## Methods

This study was conducted on 4 belugas (two 32-year-old females: F1, F2; one 27-year-old male and one 11-year-old male: M1, and M2, respectively) housed at Mystic Aquarium (Mystic, CT) in a 2.8 million liter outdoor exhibit chilled to temperatures < 16 °C. Most of the exhibit is visible from underwater through large acrylic windows. Behavioral observations were performed for one year (52 consecutive weeks) from 25 Aug 2013 to 21 Aug 2014. F2 was only available for study for the first 21 weeks of the study.

Four hours of observations were conducted per week: 2 h per week between 700 and 1000 h, and 2 h per week between 1500 and 1800 h, yielding 208 h of observation completed in 211 observation sessions lasting 30–90 min each. Continuous observations were conducted using a tripod-mounted digital video camera at an underwater viewing window. An event sampling rule was used in which the videographer focused filming on any social interaction that occurred during the filming period, in which two or more whales were within one body length (approximately 4 m), regardless of participants, resulting in a continuous record of all social interactions visible from underwater viewing. Observations were only conducted outside of training sessions. Video data used for this study were previously analyzed for the occurrence of social behaviors (Richard et al. 2021).

### Developing an ethogram for melon shape repertoire

A melon shapes ethogram was developed from the video and still images captured from the video (Table 1; for a video ethogram see electronic supplementary materials). As individual melons varied in size and structure, shapes were categorized by conserved movements that appeared to be determined by the musculature that is presumably similarly arranged in all individuals. Melon shape definitions are independent from the variable occurrence of a simultaneous open mouth behavior, which was recorded separately (see below). Melon shake was described previously (Richard et al. 2021).

**Table 1:** Melon shape ethogram

Melon shape	Representative images from a single Beluga (low-resolution frame captures from video data)	Behavior definition
No shape present		Typical melon conformation; facial musculature apparently relaxed
Melon flat		Anterior portion of the melon is compressed, reducing or eliminating the normal rounded shape of the melon’s anterior portion; although degree of compression can vary, the lateral aspects of the melon do not change shape in the transverse plane
Melon lift		Melon is raised dorsally, causing it to appear taller in the dorsal–ventral plane without shifting the melon’s leading edge anteriorly or posteriorly; results in subtle depressions on the anterolateral surfaces of the melon approximately one third of the distance from the melon’s leading edge to the eye
Melon press		Melon is flattened along the maxilla, with simultaneous prominent bulging along the maxilla, creating clear definition along the junction of the melon and the maxilla, which results in a projection of the melon anteriorly, but this shape is distinguished from a push by the more boxy appearance of the melon instead of a rounded, bulbous shape
Melon push		The melon’s anterior portion is pushed forward, emphasizing the bulbous shape of the melon’s anterior portion; often results in a dorsoventral depression along the melon’s dorsal surface approximately half way between the melon’s leading edge and the eye
Melon shake		Beluga vigorously shakes head in dorsal/ventral plane, causing the melon to shake, resulting in marked dorsoventral extension (2) and compression (3–4) of the melon (Richard et al. 2021)

Melon shape definitions are independent from the variable occurrence of a simultaneous open mouth behavior

The proposed melon shapes ethogram was then externally validated by determining if the defined melon shapes occurred in a second study population. Underwater observations of three different social groupings of belugas of both sexes, ranging in age from calf to adult, were conducted at MarineLand Canada (*n* = 51 belugas). A tripod mounted digital video camera was used to capture the greatest number of animals in frame at one time, regardless of social activity. A total of 5.5 h of video data was collected and analyzed for all occurrences of melon shapes previously defined in the ethogram that were performed by any individual (individual identification was not attempted). Further analyses (described below) were conducted using video data from the primary study population only.

### Quantifying behavior

Behavioral event frequency and state duration was quantified for all interacting whales for all social interactions observed using CowLog software (Hänninen and Pastell 2009) and continuous recording (Martin and Bateson 2007) by one of three observers using the ethogram in Richard et al. (2021). For each event, the actor and recipient were identified. The recipient of a behavior in a social interaction with more than two whales was determined by the direction of the actor’s rostrum during a behavioral event. At this stage, melon shapes were identified as having occurred, but were not categorized by shape type. A still image was captured for each occurrence of a melon shape using the “Snapshot” feature in VLC Media Player (www.videolan.org). The time stamp for the start of each shape was recorded, and the duration of the melon shape was timed to the nearest second (minimum 1 s). A single observer (IP), externally validated for coding accuracy (see below), used the screenshots to confirm the actor, recipient, and the presence of a shape, and then categorized each shape into the full ethogram if clearly visible (Table 1), referring to the video as necessary. The occurrence of any concurrent behavioral events (open mouth, mouthing, or genital present) at any time during the performance of the shape was also recorded. Fifty-four shapes (2%) did not conclusively match ethogram descriptions and were excluded from analysis. Melon shake frequency in these observations was previously reported (Richard et al. 2021), but this behavior was explored in greater detail here to measure the duration and to evaluate social use, recipient’s attention, and the behavioral context of this shape’s occurrence.

### Social use: non-social observations

Video from 93 observation sessions, representing all months, were analyzed for segments without social interaction that had a beluga in view. All occurrences of melon shapes outside of social interactions were recorded until 10 min of non-social footage for M1, M2, and F1 was observed in each observation session. Some observations did not contain 10 min of non-social video for each whale; the total non-social video collected for each whale exceeded 10 h (M1: 636 min, M2: 635 min, F1: 857 min). No behaviors directed toward humans were quantified.

### Recipient attention

Each melon shape occurring during social interaction was examined to determine if the actor was in the recipient’s line of sight at any point during the duration of the shape. The still image of each shape was used, with further additional video review for all shapes that were not in the recipient’s line of sight in the still image. Belugas have greater head mobility relative to other odontocetes and often moved their head while producing or receiving a shape. The true field of view for belugas is unknown. However, two high-resolution retinal areas are thought to enable primarily monocular panoramic vision with a region of 20–30 degrees of overlapping, potentially binocular vision ventrorostrally (Mass and Supin 2002, 2018). Here, a shape was considered to be in the line of sight of the recipient if at any point during the duration of the shape, the actor’s melon was within one body length of the recipient’s closest eye, with the actor’s head within an unobstructed field of view between approximately 120˚ posteriorly from either eye (well within the field of view of a horse, which has similarly positioned eyes [Hanggi and Ingersoll 2012]), and meeting the following conditions: (1) if it was produced by an actor swimming closely alongside the recipient (within approximately 1 m), the actor’s melon must be in a position anterior to the insertion of the pectoral flipper of the recipient, without the recipient’s head being posterior to the insertion of the pectoral flipper of the actor, (2) the actor was not oriented along the long axis of the body such that the mandibular region would obscure the melon from the recipient, and (3) the recipient’s dorsal region was not the closest surface to the actor, such that the recipient’s back was not turned to the actor (see Electronic Supplementary Materials).

### Behavioral context

For each actor-recipient dyad, each observation session was classified into behavioral context categories depending on the occurrence of specific behaviors (Table 2). All melon shapes occurring between that dyad during that observation session were assigned to that behavioral context. The occurrence of open mouth, mouthing and group swimming in multiple contexts necessitated a decision tree-based process to categorize social interactions. The presence of a genital present between male–female pairs indicated courtship, as these displays occurred almost exclusively during the breeding season, and primarily occur during female ovarian cycles in a previous study of this population (Richard et al. 2021). The presence of a genital present between males indicated socio-sexual play behavior, as the genital region is pushed toward a recipient, and may be accompanied by erections (Hill et al. 2015b; Lilley et al. 2020; Richard et al. 2021). Group swimming without genital presents indicated other affiliative behavior, as this behavior requires cooperation such that the animals match speed and direction while swimming (Hill et al. 2015b; Richard et al. 2021). Mouthing, including biting and raking behaviors observed in agonistic behavior in other odontocetes (e.g. Connor et al. 2000) indicated agonistic behavior only when it occurred in the absence of a genital present or group swimming, as this behavior also occurs in socio-sexual contexts in belugas (Lilley et al. 2020). Lastly, an open mouth behavior in the absence of a genital present, group swimming or mouthing constituted an ambiguous category, as this behavior is performed in a variety of social interactions (Hill et al. 2015b). The frequency of these behavioral indicators of context from these observations has been reported previously (Richard et al. 2021); their occurrence is presented here only to assign a behavioral context to melon shape behaviors.

**Table 2:** Ethogram of behaviors used to categorize observation sessions into a behavioral context (Richard et al. 2021)

Behavior	Definition
Genital present	Whale stops active forward progress by terminating fluke beating and drifts in the direction of another whale while arching their caudal peduncle so that the genital region is pushed closer to the recipient whale; caudal end of the caudal peduncle is correspondingly angled dorsally; rostrum is often directed toward the recipient whale for some portion of the presentation causing the body to assume an ‘S’ shape; flukes and flippers may be held at various angles to control the drift towards the recipient whale; may result in contact of the genital region with the recipient
Group swim	Two or more whales swim in the same direction at approximately the same velocity for at least 30 s; all whales are within 2 m of at least one other whale in the group; bodies can be aligned or staggered (one whale swims ahead of the other), but one whale may not be completely behind another; body orientation of individuals may vary
Mouthing	A whale contacts another whale with their open mouth
Open mouth	A whale opens mouth wide enough so that the tongue is (or would be) visible

To visually represent differences in melon shape occurrence by context, the observed frequency per minute of each melon shape type used in each behavioral context was compared to the frequency per minute of each melon shape that would be expected to occur if the number of each shape type observed was proportional to the amount of time the behavioral context was observed (null hypothesis). The proportion of the total time that each context was observed relative to the total time spent socializing was multiplied by the total number of each shape type observed in the study to calculate the expected frequency for each shape type in each context. The difference between the observed frequency and the expected frequency of each shape type was divided by the expected frequency and multiplied by 100. Differences from the expected frequencies were converted to a percent to account for the wide variation in shape type frequencies to allow more direct comparisons.

### Elaboration of melon shapes

The mean, median, and interquartile range of the shape duration was calculated for each shape type in each behavioral context. The proportion of melon shapes that occurred simultaneously with another behavior (open mouth, mouthing, or genital present) was calculated for each shape type in each behavioral context.

### Data analysis

All statistical tests were performed in R version 4.3.0 (R Core Team 2023). F2’s 3 shapes were excluded from statistical analysis. To test if melon shapes occurred at a higher rate while in social interactions than outside of social interactions, the rate per minute in each condition (*n* = 3) were compared using a permutation test in the R package permuco with an exhaustive enumeration of 720 permutations (Frossard and Renaud 2021).

To test the hypothesis that the occurrence of specific melon shape types varied by context, logistic mixed effects regression models estimated using maximum likelihood were constructed using the glmer function in the R package lme4 (Bates et al. 2015). Each of the five possible melon shape types were coded as a binary response factor for each melon shape that was classified into a behavioral context (2425 melon shapes). The fixed effect of behavioral context was coded as a categorical predictor variable. The identity of the actor was included as a random intercept term to account for observations being clustered by individual. The occurrence of each specific melon shape type was tested separately. To test the hypothesis that the elaboration of shapes will depend on the shape type and the behavioral context in which it occurs, logistic mixed effects regression models estimated using maximum likelihood were constructed using the glmer function in the R package lme4 (Bates et al. 2015). The binary response variables of duration and the occurrence of a concurrent open mouth display were tested separately. Duration was modeled as a binary response factor (greater than the median duration of the entire data set or equal to or less than the median) because there were only 19 unique values of duration and the data was highly skewed. Behavioral context and the melon shape type were coded as categorical predictor variables as individual fixed effects with a context:shape interaction term, with the identity of the actor coded as a random intercept term to account for observations being clustered by individual. For all models, the significance of the fixed effects was tested using a Wald chi-square test, and the influence of the predictors on the response variables were further assessed by constructing models with and without the fixed effect(s) and comparing model fit using analysis of variance (ANOVA), log likelihood, and Akaike information criterion (AIC). Significance was set at *p* < 0.05.

### Interobserver agreement

Interobserver agreement methods and results were reported for three observers of behavioral states and events, including melon shake but excluding other melon shapes in Richard et al. (2021). Briefly, the occurrence of interactions had 98.5% agreement. Using 10 h of video containing 9% of all social interactions, genital present, group swim, mouthing, open mouth, and melon shake had “excellent” agreement (*κ* > 0.75) with the reference observer (JR) except mouthing for one observer pairing, which had a “good” agreement (*κ* = 0.63) (Kaufman and Rosenthal 2009). The occurrence of a non-shake melon shape had “fair” agreement (*κ* > 0.5) with the reference observer; false positive identifications that reduced interobserver agreement on the occurrence of the shape would later be eliminated from analysis through the evaluation of still images. Finally, to externally validate the full melon shape ethogram, the kappa statistic was calculated using a data set of 190 randomly selected screenshots analyzed by JR and IP. Agreement was “excellent” (*κ* > 0.75) for all 4 shapes (flat: *κ* = 0.88; push: *κ* = 0.82; press: *κ* = 0.84; lift: *κ* = 0.76).

## Results

### Melon shape repertoire

A total of 2570 melon shapes were observed in the primary study population (Table 3). All 5 identified shape types were performed by F1, M1, and M2; F2 only performed melon flat but this whale was present in only 4.8% of the video containing social interactions. The defined melon shapes were not unique to belugas at Mystic Aquarium, with all 5 shapes also observed in MarineLand Canada belugas. A total of 72 melon shapes were observed at MarineLand Canada (25 flats, 6 lifts, 10 presses, 29 pushes, and 2 shakes).

**Table 3:** Melon shapes observed from the primary study animals

ID	Time spent socializing (min)	Melon shape					Total
		Flat	Lift	Press	Push	Shake	
F1	783	146	14	42	137	75	414
F2	106	3	0	0	0	0	3
M1	744	79	30	74	259	46	488
M2	1357	562	98	364	358	283	1665
Total	1437	790	142	480	754	404	2570

Time spent socializing includes interactions with 2–4 belugas, such that the sum of individual times spent socializing exceeds the total amount of time that social interactions were filmed

### Social use and recipient’s attention

**Fig. 2:**
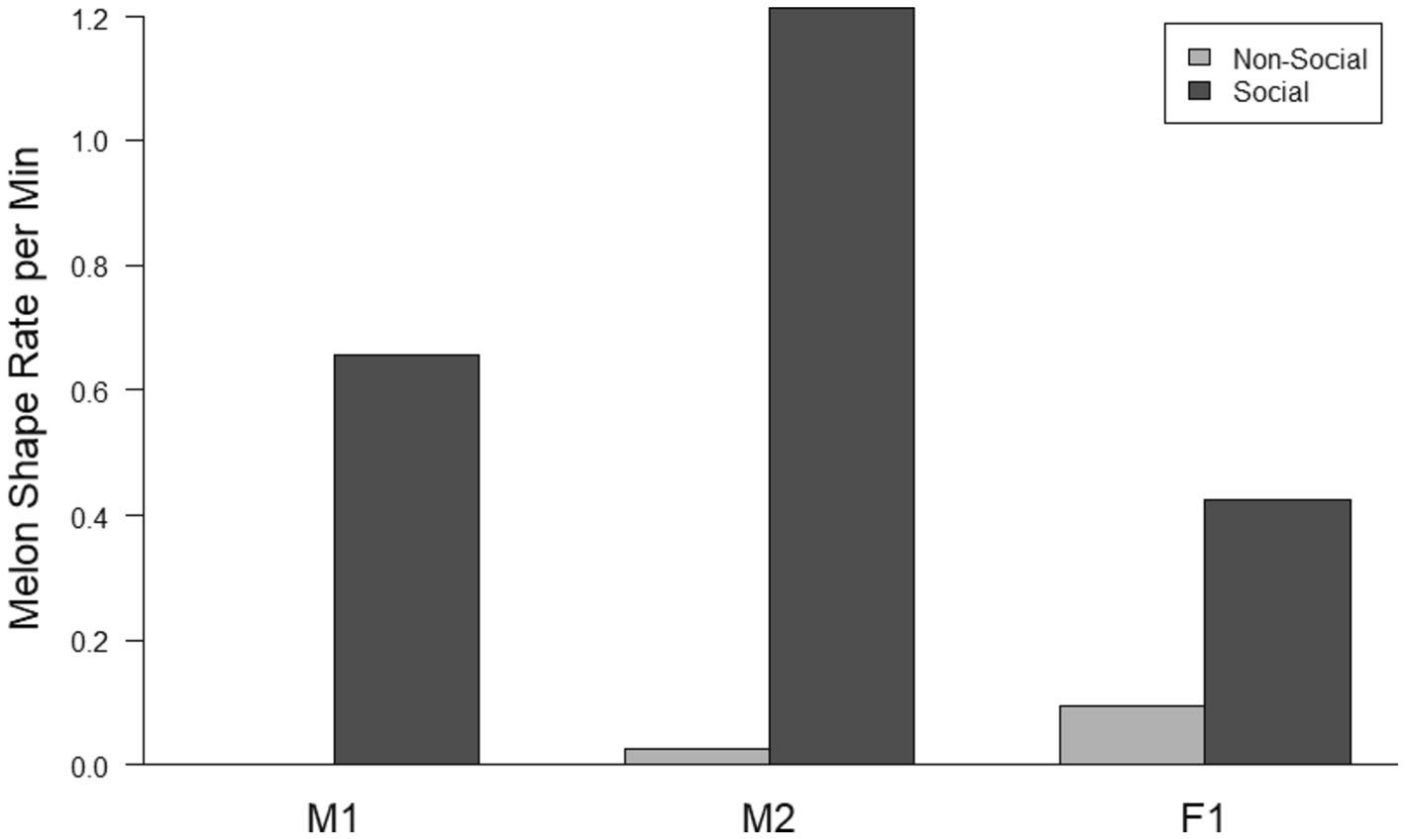
Melon shape rate per minute by individual for observations during social interactions and outside of social interactions

Melon shapes were performed more frequently during social interactions (96.1% of all shapes) than outside of social interactions (*t* = 3.06, *p* < 0.05; Fig. 2). A total of 2471 melon shapes were categorized in 1437 min of social interaction (1.72 melon shapes per minute of social interaction). A total of 99 melon shapes were observed in 2128 min of non-social video (0.05 melon shapes per minute) (Fig. 1); of these, 98% were melon flats (81 performed by F1 and 16 performed by M2). All further results will only consider the melon shapes performed during social interactions.

A total of 158 shapes (representing 6.4% of all 2471 shapes performed during social interactions) were performed out of the recipient’s line of sight. The most common reasons for a shape being classified as out of sight were violations of conditions 1 and/or 2: the recipient was swimming away from the actor (31.0%), the actor was concurrently mouthing the recipient’s body (30.3%), or the actor was swimming closely alongside the recipient, but with their head behind the insertion of the recipient’s pectoral flipper (25.3%). Violations of condition 3 (the recipient’s back was turned toward the actor) occurred less often (10.1%). M2 was more likely to perform an out of sight shape than F1 or M1 (see electronic supplementary materials).

### Behavioral context

**Fig. 3:**
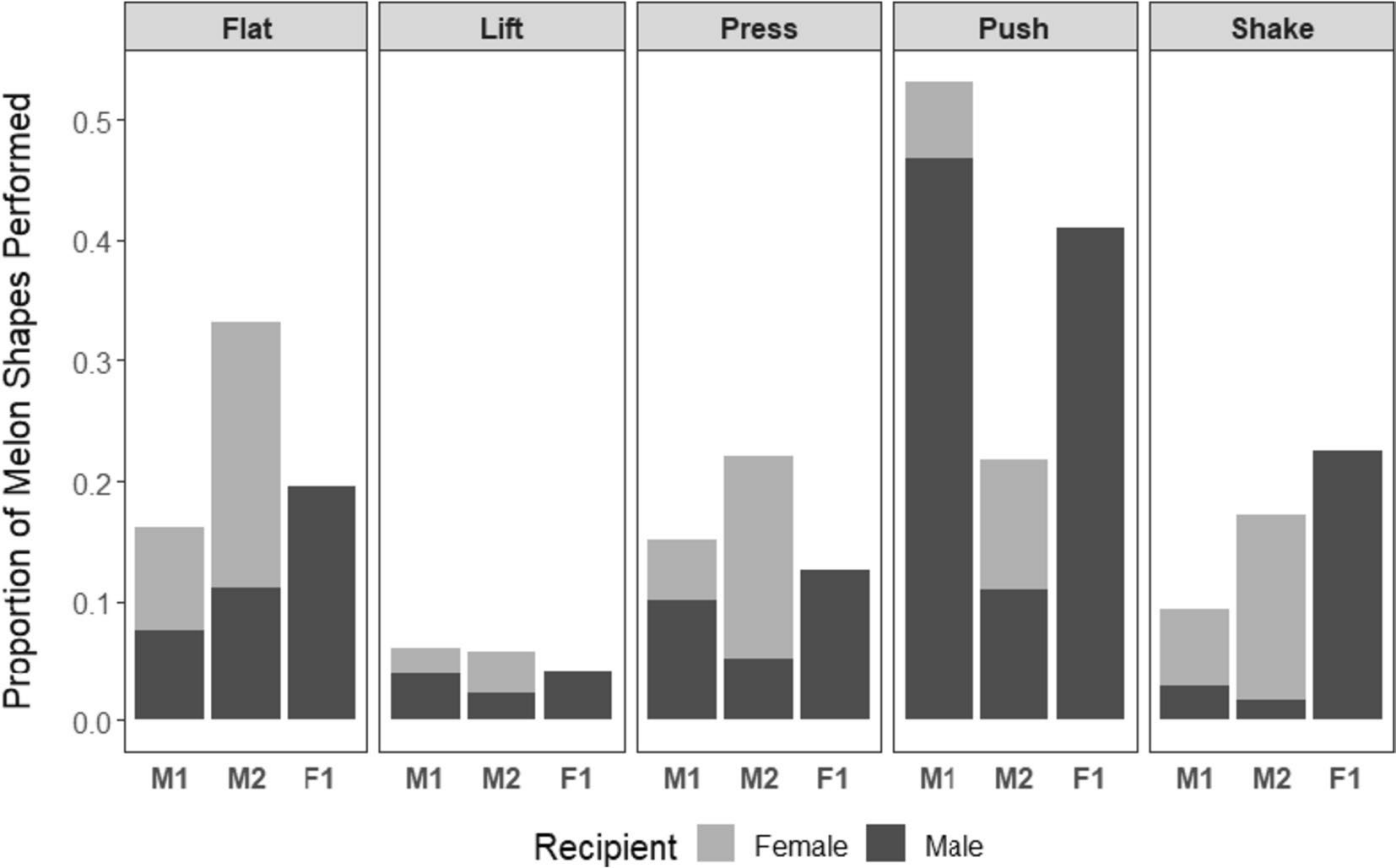
Proportion of melon shapes performed by individual. Shading is scaled to the proportion of each shape that was directed toward a male or female recipient

Males performed shapes more than three times as frequently (1.34 per min socializing with females and 1.30 per min socializing with a male) as females (0.38 per min socializing). Shape types were not observed at equal frequencies, and the proportion of occurrence varied by individual and the recipient’s sex (Fig. 3).

**Fig. 4:**
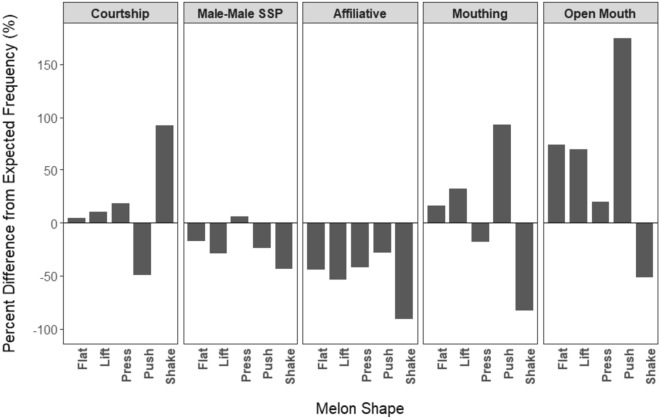
Percent difference from the expected frequency of each melon shape in each behavioral context if shapes occurred proportional to the amount of time each behavioral context was observed (*SSP* socio-sexual play). The proportion of time in each context was multiplied by the total number of each shape observed in the study to calculate the expected frequency for each shape in each behavioral context. The difference between the observed frequency and the expected frequency of each shape was divided by the expected frequency and multiplied by 100. Positive values indicate more frequent occurrences than expected, while negative values indicate less frequent occurrences than expected

Nearly all (98%) melon shapes could be assigned to one of the defined behavioral contexts (Table 4). Melon shape frequency varied by behavioral context. For press, push, and shake, behavioral context was a significant predictor for their occurrence relative to other shape types (press: *Χ*^2^ = 28.12, *p* < 0.001; push: *Χ*^2^ = 259.64, *p* < 0.001; shake: *Χ*^2^ = 196.83, *p* < 0.001). Specifically, press occurred more often in courtship and male-male sociosexual play than in mouthing or open mouth contexts. Push occurred less frequently in courtship than in all other contexts, and occurred less frequently in male-male sociosexual play than in affiliative, mouthing, and open mouth contexts. Shake occurred more frequently in courtship than in all other contexts, and occurred more frequently in male-male sociosexual play than affiliative, mouthing, and open mouth contexts (Fig. 4, see also electronic supplementary materials).

**Table 4:** Melon shape occurrence by behavioral context

Behavioral context	Proportion of duration of social interactions observed	Melon shape rate per Min	Melon shapes observed (Count)				
			Flat	Lift	Press	Push	Shake
Courtship	0.44	1.86	320	69	252	169	344
Male-male socio-sexual play	0.11	1.41	63	11	56	63	25
Affiliative	0.22	0.94	83	14	60	116	8
Mouthing	0.16	2.08	129	30	63	234	11
Open mouth	0.07	3.07	86	17	41	147	14

### Elaboration of melon shapes

**Fig. 5:**
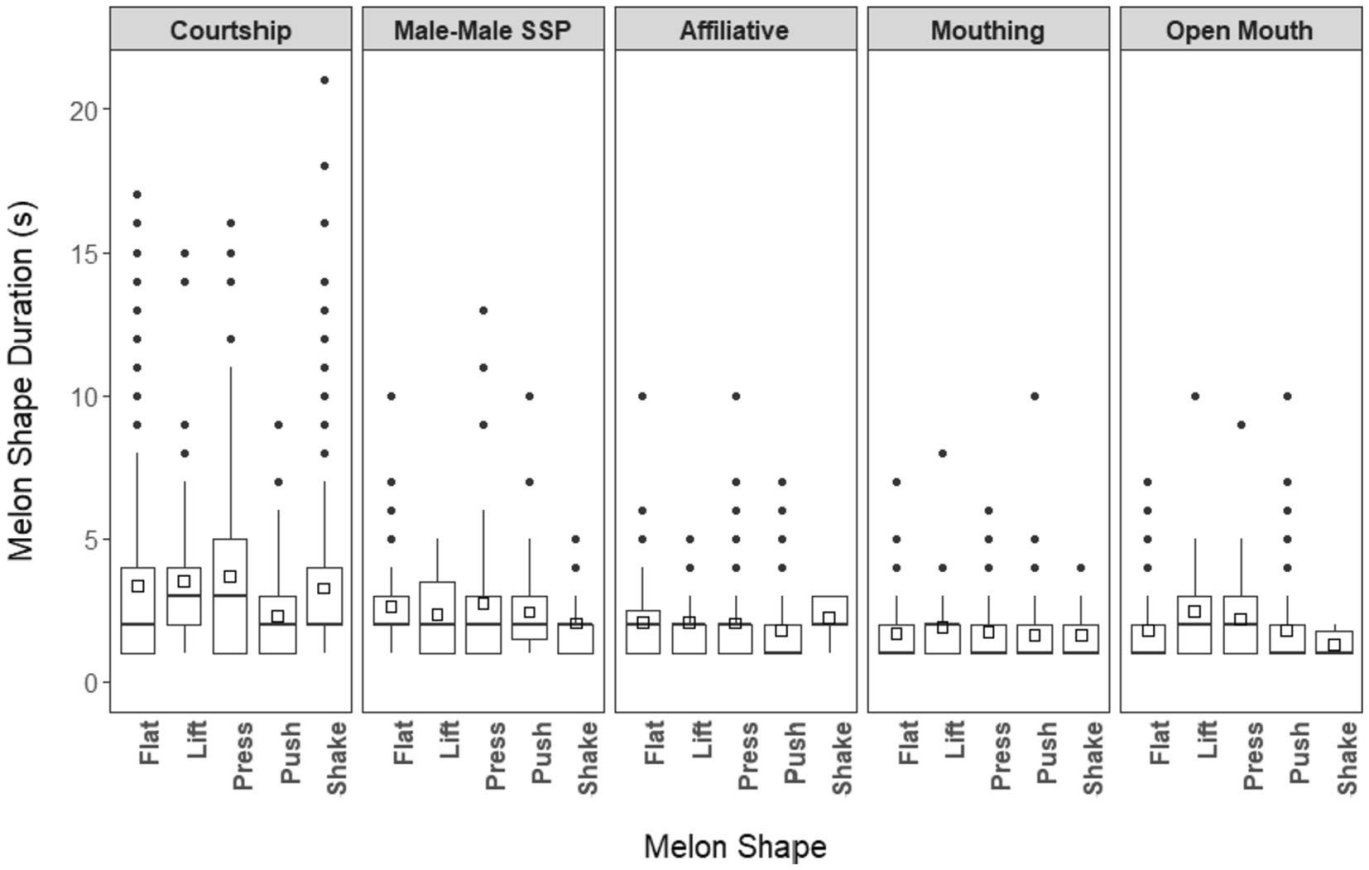
Melon shape duration by behavioral context. Open squares indicate the mean

Melon shapes lasted 2.5 ± 2.3 s (median = 2 s, range 1–21 s, IQR = 1, 3 s) (see electronic supplementary materials). Ninety two percent of shapes lasted 5 s or less and 1% lasted longer than 10 s. The occurrence of shapes with a duration longer than the median duration (2 s) was influenced by shape type (*Χ*^2^ = 10.06, *p* < 0.05) and behavioral context (*Χ*^2^ = 122.39, *p* < 0.001). Shapes with durations longer than 2 s were more likely to occur in courtship and male-male sociosexual play than all other contexts (see electronic supplementary materials). On average, shapes performed by males toward females (3.0 ± 2.7 s) and females toward males (2.6 ± 2.4 s) were longer in duration than shapes performed by males toward males (1.9 ± 1.3 s). Shapes performed during courtship (3.3 ± 2.9 s) were longer than those performed in other behavioral contexts (1.9 ± 1.4 s) (Fig. 5). Of the 203 shapes lasting longer than 5 s, 169 of them (83%) occurred during courtship, including 33 of the 35 shapes lasting longer than 10 s.

An open mouth occurred concurrently with 889 melon shapes (36% of all shapes) performed by the same actor (44% of all open mouth behaviors observed). The occurrence of a concurrent open mouth was influenced by shape type (*Χ*^2^ = 117.00, *p* < 0.001), behavioral context (*Χ*^2^ = 29.39, *p* < 0.001), and the interaction between shape type and context (*Χ*^2^ = 51.81, *p* < 0.001). The significant interaction is clearly demonstrated by observations of lift and flat with a concurrent open mouth. Compared to other shape types, lift occurred most frequently with a concurrent open mouth (71% of the time) but was nearly twice as likely to occur with a concurrent open mouth during courtship (88% of the time) than during male-male socio-sexual play (36% of the time) or mouthing (47% of the time) contexts (see electronic supplementary materials). Only 5% of flats performed during male-male socio-sexual play were accompanied by a concurrent open mouth, while 28 and 43% were during courtship and open mouthing contexts, respectively. The duration of melon shapes with a concurrent open mouth was 2.7 ± 2.4 s. Melon shapes less often occurred concurrently with mouthing (47 occurrences, 9% of mouthing events) and genital presents (17 occurrences, 4% of genital presents).

## Discussion

The near exclusive occurrence of most melon shapes in social contexts, and the varying frequency of melon shapes by individual, shape, and behavioral context suggests that melon shapes serve a communication function as opposed to a primarily echolocation-related function. Their disproportionate production while in view of a recipient also suggests that melon shapes are more accurately described as facial displays rather than facial expressions that reflect affective state. These findings are consistent with the apparent significance of non-vocal displays during social interactions in this species (Hill et al. 2015b; Richard et al. 2021; Smith et al. 1994). Although the sample size was small for this study, these findings are significant because this is the first study to explore melon shapes as communication signals, which are apparently unique to belugas among odontocetes. Their occurrence likely has been understudied to date because video recorded observations with underwater visibility are required to accurately categorize individual melon shapes. Still, these signals have the potential to provide a study system for intentional communication in cetaceans because unlike vocal communication, melon shapes are readily attributable to specific individuals.

### Repertoire

This study identified five facial displays involving the melon in belugas, four of which must require actions of facial musculature associated with the melon, while melon shake requires these muscles to be relaxed to allow the marked dorsoventral compression and extension that occurs during this behavior. This repertoire was consistent for both sexes and both populations studied. F2’s limited repertoire was likely due to the limited observations available, which meant that she was not observed in all behavioral contexts; no courtship behavior was directed toward her. However, individual variation in facial display repertoire is observed in primates (Florkiewicz et al. 2018; Molesti et al. 2020) and may explain variation in beluga melon shape use as well.

The shapes that were defined in this study appear to correspond well to the anatomy of the facial musculature reported in dolphins and porpoises. Huggenberger et al. (2009) suggest that the lateral and medial rostral muscles would act to pull the lateroventral parts of the melon ventrally to the rostrum (corresponding to melon press), the dorsal part of the melon would be pulled caudally by the intermedius muscle (corresponding to melon flat), while the anterointernus muscle would pull the caudal portion of the melon ventrally (corresponding to melon push). The melon lift is less clearly explained, but the depressions created on either side of the melon during this display suggest that musculature is compressing the melon medially. This repertoire suggests that the lipid portion of the melon is displaced by muscle movements. In contrast with other odontocetes with bulbous melons, such as the pilot whale, the bulbous shape of the beluga melon is accomplished without a thickening of dermal connective tissue superficial to the melon (Mead 1975), perhaps allowing the observed mobility.

Although the shapes defined in this study were consistent across individuals and correspond well to odontocete facial musculature, this repertoire may be an underestimation of the full repertoire of melon shapes used by belugas. Contexts important to the ecology of these animals that might require different communication signals, such as maternal care or foraging, were not observed in this study. Our limited sample size precludes species-level generalizations, especially with the absence of young animals, which may use broader gestural repertoires than adults (Aychet et al. 2021; Molesti et al. 2020) and are known to diversify the behavioral repertoire of beluga social groups (Hill et al. 2015a). There are also ontogenetic changes to the beluga’s head shape, with calves having a more blunt, porpoise-like appearance (Brodie 1989) that may restrict their capacity for producing melon shapes. Longitudinal studies of the development of socio-sexual behavior (Lilley et al. 2020) and vocal behavior (Vergara and Barrett-Lennard 2008) have revealed social influences on the ontogeny of these behaviors. Similarly, longitudinal studies in aquaria could reveal if melon shapes develop over time, or if developmental factors influence the repertoire of melon shapes used by adults.

Documentation of the melon shape repertoire was also limited by the ability to accurately classify melon shapes through visual observation. Individual differences in melon size and shape complicate melon shape recognition. Additionally, like primate facial expressions, melon shapes are graded signals; they have an onset, reach an apex that can vary in intensity, and then release back to the relaxed position (Waller and Micheletta 2013). Varying intensity resulted in subtle variations in the shapes observed in this study. Melon flat especially appeared to have varying intensity, with shape-to-shape differences in how far caudally the anterior portion of the melon was retracted. Attempting to quantify these gradations would likely lead to reduced interobserver agreement (Waller and Micheletta 2013), and it is unclear if these gradations are meaningful communication signals that justify further classification. Melon flat had the weakest association with behavioral context, perhaps reflecting unclassified yet meaningful variations that obscured these associations. A deeper knowledge of facial musculature in a variety of mammals has led to the development of a more rigorous method to classify these gradations (reviewed in Waller et al. 2020); a similar method for belugas may reveal a larger repertoire than what has been documented here.

### Social use and recipient attention

Melon shapes were disproportionately performed in social contexts while in view of a recipient, two conditions used to identify potentially intentional communication in other species (Waller et al. 2015). This suggests that rather than being performed reflexively, the actor has voluntary control over muscles that produce melon shapes, as has been suggested for facial displays in several primate species (Aychet et al. 2021; Scheider et al. 2016; Waller et al. 2015) and demonstrated in this species by training melon shape changes on cue (Fig. 1). This also implies that if melon shapes function primarily during echolocation, belugas would be able to voluntarily change their melon’s shape to adapt echolocation signals to the environment or current task. However, if changing the melon’s shape primarily served an echolocation-related function, more shapes might be expected to occur in contexts in which echolocation is presumably more useful for gathering sensory information, such as when a social partner is swimming away or when other belugas are more than one body-length away. The sheer preponderance of these displays during interactions occurring in close proximity, in full daylight and in clear water, further implies their communicative function. Still, they could serve both functions, or some melon shapes may function primarily in either communication or echolocation. The lack of acoustic recordings during this study precludes these determinations. Belugas are well-known for their diverse vocal repertoire (Garland et al. 2015); it is possible that changing their melon’s shape changes the acoustic properties of communicative vocalizations. Simultaneous acoustic recordings and video observations in all lighting conditions are needed to resolve this question for the function of beluga melon shapes.

Even though melon shapes occurred frequently during social interactions, these observations likely represent an underestimate of the true frequency of these behaviors. Despite ideal observation conditions, the melons of socializing whales were often oriented away from the camera or were obscured by other belugas or structural supports of the underwater viewing windows. The fair interobserver agreement suggests that melon shapes are more difficult to detect than other behaviors. While false positive identifications would have been removed during the still image analysis stage, false negative identifications would remain undetected. Occasionally, it would be apparent that the beluga was producing a melon shape, but a clear view of a sufficient proportion of the head to identify the specific shape could not be achieved. However, characteristics of each shape were such that they were readily identifiable by human observers from various angles, a feature that likely relates to their effectiveness as visual signals with conspecifics.

### Behavioral context and elaboration

Variation in shape frequency and distribution of shape use by actor, recipient, and behavioral context suggests that different melon shapes convey different signals. The increased use of melon shapes by males relative to females and within male–female interactions versus male-male interactions is consistent with the production of other visual displays in belugas (Richard et al. 2021) and facial displays in some primates (Aychet et al. 2021; Lazow and Bergman 2020). However, a rigorous comparison between sexes is not possible due to the limited number of study subjects and the lack of observed female-female social interactions. This finding is also influenced by the disproportionate number of shapes performed by M2, who spent the most time socializing while also performing the most melon shapes on a rate basis. This high rate might be due to individual variation or perhaps an observation bias resulting from the morphology of his melon. M2’s younger age may also be a factor; in chimpanzees (*Pan troglodytes*), juveniles spend more time gesturing and produce more unreciprocated gestures (Hobaiter and Byrne 2011). The small sample size also precluded the use of more complicated error structures when modeling the effect of context on shape, increasing the risk of type 1 error in this study. While the clustering of observations within individual was accounted for in statistical analyses, shape use was also clustered by actor-recipient dyad and by interaction; perhaps some shapes would be more likely to be exchanged between specific pairings of individuals or within a given interaction, independent of context. A larger sample size is needed to explore these potential influences on melon shape use by individuals across behavioral contexts.

Courtship was associated with higher-than-expected rates of melon shapes, particularly melon shake. The relatively high rate of melon shake in courtship contexts has been noted previously (Richard et al. 2021), with this study demonstrating the relatively infrequent occurrence of this shape in other contexts. The relatively high rate of melon shapes, especially when contrasted with other affiliative contexts, is consistent with the importance of other visual displays during courtship (Richard et al. 2021). Melon shapes performed within the courtship context were longer in duration than shapes performed in other contexts, suggesting an elaboration of the communication signal. Primates elaborate facial displays through more intense muscle movements, longer durations, or by combining displays (Aychet et al. 2021; Lazow and Bergman 2020; Waller et al. 2015), which then affect the recipient’s attentional state or response. Lengthening the display duration may function to ensure the signal’s transmission to the recipient, which might have greater fitness consequences in a reproductive context.

Relatively high rates of melon shapes also occurred during mouthing and open mouth interactions, but with distributions of shape use that were dissimilar from courtship. The contrasting use of melon push in mouthing and open mouth contexts relative to affiliative contexts is particularly notable. Unlike melon flat and melon press, melon push results in the melon looking larger in size than in the relaxed state. Perhaps this shape serves to make the actor appear larger to the recipient, a common characteristic of threat displays across taxa (Számadó 2008). Melon flat and melon lift were not associated with specific behavioral contexts, perhaps indicating more flexible usage. The varied elaboration of these shapes with a concurrent open mouth across behavioral contexts suggests modulation of the intended signal, allowing the same shape to be used for different communication purposes. Alternatively, the relatively more graded nature of melon flat or the broad behavioral context definitions used in this study prevented the detection of associated behavioral context for these shapes.

The relative frequency of melon shapes used in the open mouth context compared to others supports the current ambiguity of the open mouth behavior’s function in belugas in the absence of other context-indicating behaviors. Although the open mouth behavior is a threat display used in agonistic interactions by belugas and other odontocetes (e.g. Overstrom 1983), it is also frequently performed by belugas engaged in affiliative interactions, such as courtship and socio-sexual play (Lilley et al. 2020; Richard et al. 2021). It is reasonable to assume that there must be other behavioral elements that modulate this commonly used signal, such as concurrent vocalizations or variation in the duration or gape angle of the open mouth. Melon shapes may also enhance the open mouth display, similar to how geladas (*Theropithecus gelada*) utilize the optional lip flip signal intensifier with the bared teeth display to communicate benign intent (Lazow and Bergman 2020). Indeed, the highest rate of concurrent open mouth behaviors with a melon shape occurred in the open mouth behavioral context, even though the open mouth behavior was commonly observed across all contexts. The type of shape, as well as the timing of shape performance relative to the mouth opening (starting or ending simultaneously with, before, or after the open mouth) may modulate the signal intended by the open mouth, the melon shape, or both.

### Exclusivity among odontocetes

The diverse repertoire of facial displays produced by belugas in social contexts begs the question of why belugas should be unique among the odontocetes in their ability to create such displays. Living in an aquatic environment and spending significant time at depth in low light conditions would theoretically make visual signals, particularly subtle signals, less effective (Huggenberger et al. 2009). This is especially true for belugas, which are known for living at high latitudes where sunlight is limited for much of the year. The clear water conditions in aquaria may cause individuals to emphasize the use of these displays when communicating relative to wild belugas, and perhaps insufficient underwater observations have been made of all odontocetes to know if melon shapes are truly unique to belugas. However, there are several features of beluga natural history that may increase the adaptive significance of visual signaling relative to other odontocetes.

One predictor of complex communication is large social group size. In a study of 12 non-human primate species, the repertoire of facial movements is positively correlated to the mean group size (Dobson 2009). Beyond group size, other factors such as group density, the linearity of the dominance structure in a group, and the number of group member roles is predicted to increase communication complexity (Freeberg et al. 2012). Beluga social groups are not matrilineal, but rather may be considered communities numbering in the hundreds or thousands that consist of a variety of social groupings that contain a mix of kin and non-kin, with memberships that can be fluid depending on social or ecological variables (O’Corry-Crowe et al. 2020). In concert with their diverse vocal repertoire, the beluga’s facial mobility increases the complexity of their communication repertoire, perhaps facilitating the maintenance of their complex social structure.

Short range visual communication could be important for belugas during courtship and mating. Unlike all other odontocetes studied to date, beluga ovulation is induced by copulation (Steinman et al. 2012). This ovulation mode, relative to spontaneous ovulation, can influence mating strategies because the first male to copulate with a receptive female typically sires the offspring (Soulsbury 2010; Soulsbury and Iossa 2010). Female belugas would be expected to employ precopulatory mate choice, which could occur through evaluation of the frequent and vigorous visual displays performed by males, which prompt varying behavioral responses from the female (Richard et al. 2021). As suggested by the rate of melon shapes in courtship contexts in this study, melon shapes may be an additional visual display that females use to evaluate potential mates. This is further supported by the anecdotal sexual dimorphism of the beluga melon, with older males possessing more prominent melons that almost “overhang” the rostrum in some individuals (Kleinenberg et al. 1969). If present, this sexual dimorphism of the melon would exist despite an apparent lack of sexual dimorphism of the skull (Vicari et al. 2022). Larger melons would presumably increase the visibility and potential elaboration of melon shapes, enhancing these displays. The lipid in the melon is an irreversible energy investment (Koopman et al. 2003); perhaps melon size represents an honest indicator of male quality which is then emphasized by melon shapes. The visibility of these displays would be enhanced by the white coloration of adult belugas providing contrast in the marine environment, perhaps explaining the unique and characteristic coloration of this species. A more complete description of the melon’s sexual dimorphism, as well as any ontogenetic changes in morphology, especially in males, is required to evaluate these potential functions.

### Conclusion

Melon shape facial displays are a diverse and frequently occurring behavior used in social contexts while in view of a recipient. They are graded signals that can be elaborated through intensity and duration through apparent voluntary control of the facial musculature. They are optionally produced concurrently with an open mouth, perhaps to modulate the intended signal. Their varied performance in frequency and shape type depending on actor, recipient, and behavioral context support a communicative function as opposed to reflexive responses to affective state or movements intended to enhance echolocation. Although significant questions remain due to the small sample size and limited behavioral and social contexts observed in this study, these findings suggest that melon shapes carry some of the indicators of intentional communication. Therefore, belugas provide a valuable study system for this line of research, particularly in aquaria, where behaviors are more readily observed and can be placed within the context of the known social composition of the group as well as the age, sex, reproductive state, and developmental history of each individual.

## Supplementary Information

Below is the link to the electronic supplementary material.

Supplementary file1 (MP4 88860 KB)

Supplementary file2 (PDF 205 KB)

## Data Availability

The datasets generated during and/or analyzed during the current study are available from the corresponding author on reasonable request.
