# Supplementary material for: Belugas (Delphinapterus leucas) create facial displays during social interactions by changing the shape of their melons
Source: Anim Cogn. 2024 Mar 2;27(1):7. doi: 10.1007/s10071-024-01843-z (PMC10907495; doi:10.1007/s10071-024-01843-z)
Supplement: Supplementary file 2 — Supplementary file2 (PDF 205 KB) [file 10071_2024_1843_MOESM2_ESM.pdf]

Supplementary figures and tables for:

Belugas (*Delphinapterus leucas*) create facial displays during social interactions by changing the shape of their melon

Justin T. Richard[1]*, Isabelle Pellegrini[1], and Rachael Levine[1]

[1]Department of Fisheries, Animal and Veterinary Science, University of Rhode Island, Kingston, RI 02881, USA

*Corresponding author: jt_richard@uri.edu

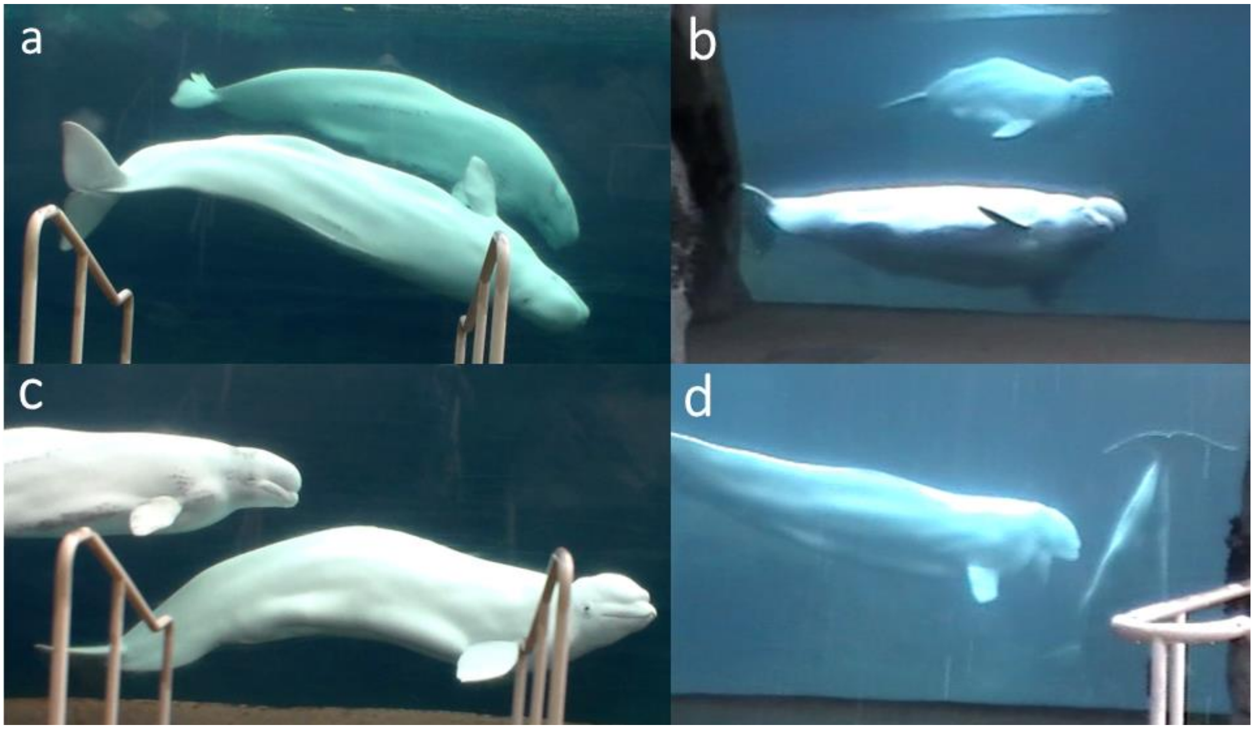

Fig S1 Examples of melon shapes occurring out of the field of view of the recipient: a) actor's ventral surface facing recipient; b) recipient's dorsal surface facing actor; c) close alongside with actor's melon anterior to the pectoral flippers of the recipient; d) close alongside with the actor's melon posterior to the pectoral flippers of the recipient.

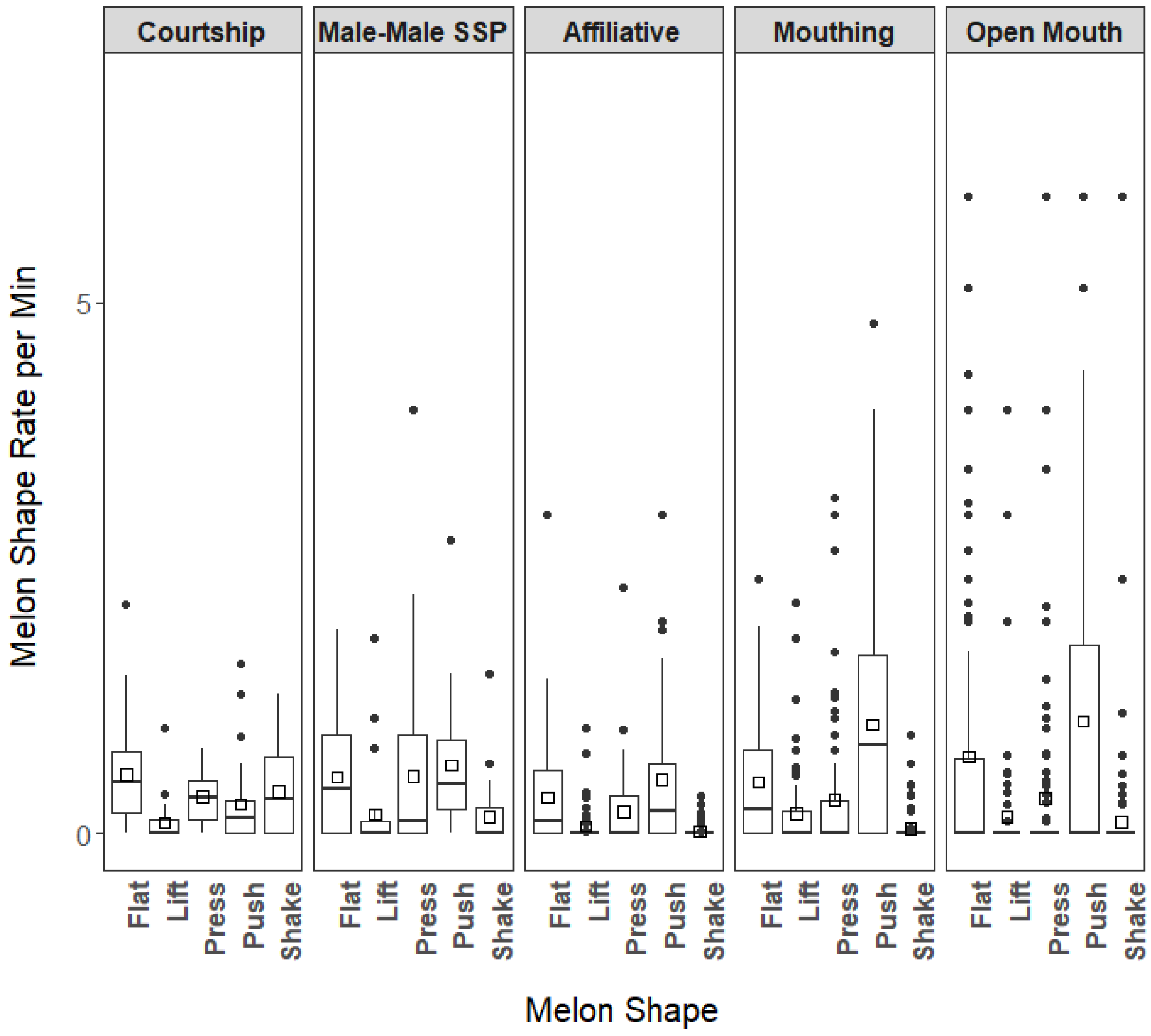

Fig S2 Melon shape rate per minute within individual dyadic interactions assigned to each context (sum of melon shape occurrences of both animals in the interaction, divided by the duration of the interaction in minutes). Open squares indicate the mean. Seven outlying values > 7 are not shown on this plot (2 in Mouthing, 5 in Open Mouth).

Table S1 Actor and shapes performed out of the recipient's line of sight relative to the total amount of shapes performed by that actor, and the distribution of shape type

	**Proportion of All Shapes**	**Proportion of Shapes Performed Outside Line of Sight (158 total occurrences)**	**Percent Difference from Expected Frequency**
**ID**			
F1	0.13	0.04	-67%
M1	0.20	0.15	-25%
M2	0.67	0.81	21%
**Melon Shapes**			
Flat	0.28	0.32	12%
Lift	0.06	0.10	77%
Press	0.19	0.20	4%
Push	0.30	0.33	8%
Shake	0.16	0.05	-69%

Table S2 Model selection summary for determining the effect of behavioral context on the occurrence of each melon shape type

**Shape**	**Model**	**AIC**	**Log Likelihood**	**ANOVA Results**
**Flat**	Full	2826.5	-1407.3	Not significantly different
	Intercepts only	2819.6	-1407.8	
**Lift**	Full	1086.5	-537.23	Not significantly different
	Intercepts only	1079.5	-537.76	
**Press**	Full	2356.5	-1172.3	Full model significantly better ($p < 0.001$)
	Intercepts only	2378.2	-1187.1	
**Push**	Full	2480.5	-1234.2	Full model significantly better ($p < 0.001$)
	Intercepts only	2796.1	-1396.0	
**Shake**	Full	1863.8	-925.88	Full model significantly better ($p < 0.001$)
	Intercepts only	2164.3	-1080.13	

Table S3 Significant contrasts in logistic mixed effects regression models for the effect of behavioral context on the occurrence of each shape type. Behavioral contexts are coded numerically (1: courtship, 2: male-male sociosexual play, 3: affiliative, 4: mouthing, 5: open mouth). Adjusted p values are reported

Shape	Contrast	Estimate	SE	z	p
Press	1:4	-0.62	0.16	-3.95	< 0.001
	1:5	-0.61	0.18	-3.30	0.008
	2:4	-0.81	0.21	-3.91	< 0.001
	2:5	-0.79	0.23	-3.44	0.005
Push	1:2	1.14	0.20	5.79	< 0.001
	1:3	1.92	0.17	11.30	< 0.001
	1:4	2.18	0.15	14.42	< 0.001
	1:5	2.00	0.16	12.30	< 0.001
	2:3	0.78	0.20	3.84	0.001
	2:4	1.04	0.18	5.68	< 0.001
	2:5	0.86	0.20	4.34	< 0.001
Shake	1:2	-1.19	0.22	-5.33	< 0.001
	1:3	-2.68	0.37	-7.33	< 0.001
	1:4	-2.87	0.32	-9.16	< 0.001
	1:5	-2.18	0.28	-7.75	< 0.001
	2:3	-1.49	0.42	-3.57	0.003
	2:4	-1.68	0.37	-4.51	< 0.001
	2:5	-0.99	0.35	-2.86	0.032

Table S4 Duration (seconds) of each shape type in each behavioral context.

Melon Shape	Mean Duration ± Standard Deviation	Maximum Duration	Courtship Duration	Male-Male SSP Duration	Affiliative Duration	Mouthing Duration	Open Mouth Duration
Flat	2.6 ± 2.6	17	3.34 ± 3.32	2.62 ± 1.61	2.07 ± 1.55	1.70 ± 1.06	1.78 ± 1.19
Lift	2.8 ± 2.3	15	3.51 ± 2.71	2.36 ± 1.37	2.07 ± 1.16	1.90 ± 1.40	2.47 ± 2.25
Press	3.0 ± 2.6	16	3.68 ± 2.94	2.73 ± 2.38	2.05 ± 1.62	1.73 ± 1.10	2.17 ± 1.58
Push	1.9 ± 1.3	10	2.30 ± 1.49	2.43 ± 1.51	1.78 ± 1.16	1.63 ± 1.03	1.80 ± 1.30
Shake	3.0 ± 2.7	21	3.26 ± 2.78	2.04 ± 1.31	2.25 ± 0.66	1.64 ± 0.98	1.29 ± 0.45

Table S5 Model selection summary for determining the effect of behavioral context and shape type on the occurrence of shapes with durations longer than the median duration (2 s)

Model	AIC	Log Likelihood	ANOVA Results
Full	2894.4	-1421.2	
Drop interaction term	2883.1	-1431.5	Not significantly better than full model
Drop context fixed effect	3024.2	-1506.1	Full model is significantly better ($p < 0.001$)
Drop shape type fixed effect	2884.1	-1436.0	Not significantly better than full model
Intercepts only	3084.4	-1540.0	Full model is significantly better ($p < 0.001$)

Table S6 Significant contrasts for behavioral contexts from logistic mixed effects regression model for shape duration. Behavioral contexts are coded numerically (1: courtship, 2: male-male sociosexual play, 3: affiliative, 4: mouthing, 5: open mouth). Adjusted p values reported

Contrast	Estimate	SE	z	p
1:3	-0.78	0.28	-2.81	0.005
1:4	-1.33	0.27	-5.00	< 0.001
1:5	-1.27	0.31	-4.13	< 0.001
2:3	-0.75	0.36	-2.08	0.037
2:4	-1.30	0.35	-3.71	< 0.001
2:5	-1.23	0.38	-3.23	0.001

Table S7 Model selection summary for determining the effect of behavioral context and shape type on the occurrence of a concurrent open mouth display

Model	AIC	Log Likelihood	ANOVA Results
Full	3001.7	-1474.8	
Drop interaction term	3022.4	-1501.2	Full model is significantly better ($p < 0.001$)
Drop context fixed effect	3051.0	-1519.5	Full model is significantly better ($p < 0.001$)
Drop shape type fixed effect	3195.7	-1591.9	Full model is significantly better ($p < 0.001$)
Intercepts only	3225.0	-1610.5	Full model is significantly better ($p < 0.001$)

Table S8 Proportion of melon shapes with a simultaneous open mouth by behavioral context.

Behavioral Context	Proportion of Shapes with a Concurrent Open Mouth					
	**All Shapes**	**Flat**	**Lift**	**Press**	**Push**	**Shake**
Female-Male	0.51	0.37	1.00	0.48	0.72	0.18
Male-Female	0.35	0.24	0.83	0.29	0.41	0.39
Male-Male	0.32	0.20	0.50	0.36	0.35	0.21
Courtship	0.41	0.28	0.88	0.34	0.66	0.35
Male-Male SSP	0.20	0.05	0.36	0.34	0.16	0.28
Affiliative	0.28	0.18	0.64	0.25	0.34	0.25
Mouthing	0.33	0.17	0.47	0.35	0.40	0.18
Open Mouth	0.43	0.43	0.71	0.34	0.44	0.21
All Occurrences		0.24	0.71	0.33	0.43	0.37
